# Development of accurate temperature regulation culture system with metallic culture vessel demonstrates different thermal cytotoxicity in cancer and normal cells

**DOI:** 10.1038/s41598-021-00908-0

**Published:** 2021-11-02

**Authors:** Chikahiro Imashiro, Haruka Takeshita, Takashi Morikura, Shogo Miyata, Kenjiro Takemura, Jun Komotori

**Affiliations:** 1 Institute of Advanced Biomedical Engineering and Science, Tokyo Women’s Medical University, Tokyo, 162-8666 Japan; 2 Department of Mechanical Engineering, Keio University, Yokohama, 223-8522 Japan

**Keywords:** Cancer therapy, Biomedical engineering

## Abstract

Hyperthermia has been studied as a noninvasive cancer treatment. Cancer cells show stronger thermal cytotoxicity than normal cells, which is exploited in hyperthermia. However, the absence of methods evaluating the thermal cytotoxicity in cells prevents the development of hyperthermia. To investigate the thermal cytotoxicity, culture temperature should be regulated. We, thus, developed a culture system regulating culture temperature immediately and accurately by employing metallic culture vessels. Michigan Cancer Foundation-7 cells and normal human dermal fibroblasts were used for models of cancer and normal cells. The findings showed cancer cells showed stronger thermal cytotoxicity than normal cells, which is quantitatively different from previous reports. This difference might be due to regulated culture temperature. The thermal stimulus condition (43 °C/30 min) was, further, focused for assays. The mRNA expression involving apoptosis changed dramatically in cancer cells, indicating the strong apoptotic trend. In contrast, the mRNA expression of heat shock protein (HSP) of normal cells upon the thermal stimulus was stronger than cancer cells. Furthermore, exclusively in normal cells, HSP localization to nucleus was confirmed. These movement of HSP confer thermotolerance to cells, which is consistent with the different thermal cytotoxicity between cancer and normal cells. In summary, our developed system can be used to develop hyperthermia treatment.

## Introduction

Despite numerous efforts have been made for ages, cancer is still one of the leading causes of death^1^. As the main cancer treatments, surgery, chemotherapy, and radiation therapy are generally performed^2–4^. However, each strategy has its own problems to be resolved, such as invasiveness or side effects. In terms of options for cancer therapy with low invasiveness and low side effects, hyperthermia has attracted significant attention to tackle this enemy of mankind^5^. It has been reported that normal cells and cancer cells differ in their tolerance of thermal stimulus^6^. Hyperthermia exploits this difference, with the diseased area being exposed to thermal stimulus. Because the thermal cytotoxicity in cancer cells is stronger than that of normal cells even with the same thermal stimulation, cancer cells can be selectively killed. However, to move this therapy beyond laboratory, the thermal cytotoxicity in cancer and normal cells should be quantitatively and systematically evaluated. Hyperthermia treatment can be combined with other therapy methods such as chemotherapy, since a reaction of a cell to thermal stimulation affects the chemical reaction or vice versa^7^. However, in this study, we investigate the direct thermal cytotoxicity as a first step.

**Figure 1:**
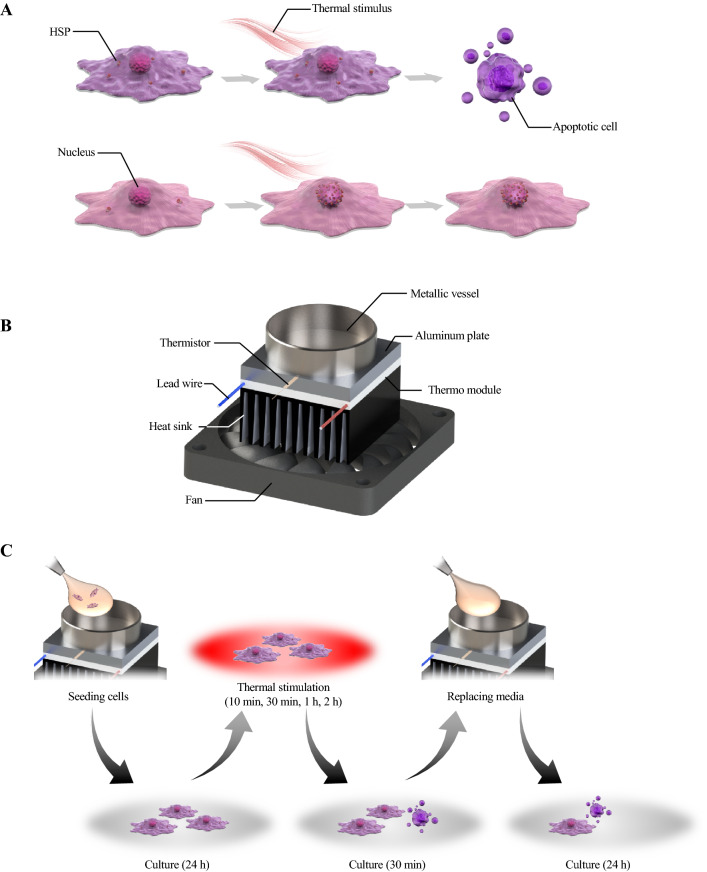
Schematic images showing the concept, the developed device, and the experimental procedure of the present work. Conceptual image of responses of cells to thermal stimulation (**A**), an overview of the thermal stimulus device in which thermal stimulation is applied to cells cultured in a metallic culture vessel having a diameter of 35 mm (**B**), and the experimental procedure to apply a thermal stimulus to cells (**C**).

Against this background, several studies have applied thermal stimulus to cells in vitro to investigate effective conditions for hyperthermia^8^. Such studies demonstrated that the intensity and duration of thermal stimulus affect the viability of cells and that cancer cells are less thermotolerant than normal cells. Furthermore, heat shock protein (HSP) has been identified as one of the key proteins conferring the difference in thermotolerance between cancer and normal cells. HSP is produced when cells are exposed to stresses such as thermal stimulus, which then localizes in the nucleus to maintain cell viability exclusively in normal cells. Interestingly, although the expression of HSP in cancer cells initially tends to be higher than that in normal cells, its specific localization is not as clear as in normal cells^9^. Figure 1A describes the different motions of HSP and different reactions to thermal stimulation between cancer and normal cells.

Despite these reports, hyperthermia treatment remains of limited use in a clinical context for treating cancer. One of the reasons for this is the lack of a database with precise information on the thermotolerance of each cell type. In most previous studies, thermal stimulus was applied to cells by controlling the set temperature of an incubator where plastic culture vessels were located^8, 10^ or of a water bath into which culture vessels were immersed^11^. This resulted in a difference between the set temperature and the actual temperature to which cells were exposed. In contrast, with an accurate temperature regulation system, we can properly investigate the thermal cytotoxicity of each cell type, which should lead to improvements in the efficacy of hyperthermia treatment. Thus, to achieve effective hyperthermia, there is a need to develop a cell culture system that is capable of regulating culture temperature immediately and accurately. Once such a system is developed, in addition to single cells, tumor models may be possible to be cultured in it.

To realize an accurately regulated thermal stimulation device, we fabricated a culture device with a metallic culture vessel and a Peltier element that can control the temperature of the culture vessel as shown in Fig. 1B. With this device, we cultured Michigan Cancer Foundation-7 (MCF-7) cells and human lung cancer cell line (A549) as a model of cancer cells and cultured normal human dermal fibroblasts (NHDFs) and human umbilical vein endothelial cells (HUVECs) as model of normal cells, respectively. Then, they were exposed to thermal stimulation to reveal the thermal cytotoxicity in each cell type. The results showed the effective conditions of thermal stimulus for hyperthermia, which may be useful for improving hyperthermia treatment. We also observed the different reactions of cancer cells and normal cells to the thermal stimulus. Our developed system for investigating the effective thermal conditions for hyperthermia should be helpful for optimizing the condition of hyperthermia treatment.

## Materials and methods

### Design of the cell culture system

**Figure 2:**
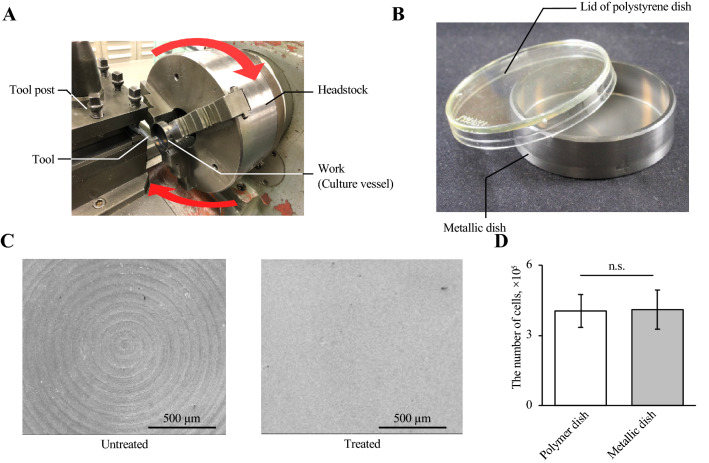
Fabrication and evaluation of the metallic cell culture vessel. Counterboring was performed on a stainless steel cylinder using a lathe for fabricating cell culture vessels (**A**). An overview of the metallic cell culture vessel (**B**). Images of cell culture surfaces with and without FPP (**C**). Comparison of the cell proliferation rate in a conventional polymer dish and the metallic vessel (*n* = 4, mean ± SD).

The cell culture system was fabricated with a metallic culture vessel and a temperature regulation component, which was composed of an aluminum plate, a thermistor, a Peltier element-based thermal module (T1406-3600; Ferrotec Holdings Corporation, Tokyo, Japan), a heat sink (15PB054-01050; Global Electronics Corporation, Tokyo, Japan), and a fan (YDH9225C12F; Shenzhen YCCFAN Technology Co., Ltd., Shenzhen, China). For the fabrication of the metallic culture vessel having a diameter of 35 mm, a process of counterboring was performed for a cylinder of stainless steel (316L) (see Fig. 2A and B). After the counterboring, fine particle peening (FPP) was performed with the conditions shown in Table S1 to vanish the processing marks and realize the adequate cell culture surface^12, 13^. The temperature regulation component was connected to a temperature regulator (VTH-1800FA; Vics Inc., Tokyo, Japan) and proportional integral control was performed for temperature regulation. Note that P and I terms are given as 10.8% and 200 s, respectively.

### Evaluation of the culture surface

The surface of the metallic cell culture vessel was evaluated by morphological observation and cross-sectional profile analysis using a scanning electron microscope (Inspect S50; Thermo Fisher Scientific Inc., Waltham, MA, USA) and a laser microscope (VK-X100; Keyence Co., Osaka, Japan).

### Measuring the temperature history

The temperature histories of the metallic culture vessel and a 35-mm conventional polymer dish (3000-035; Iwaki Co., Ltd., Tokyo, Japan) were measured with the thermistor, which was directly adhered to the culture surface with thermal adhesive tape (9885; 3 M Japan Limited, Tokyo, Japan) as shown in Fig. S1. While experimental set up shown in Fig. 1B was used for temperature regulation of the metallic culture vessel, the set temperature of the incubator was modulated for conventional polymer dish. Further, the result of temperature measurement was plotted at every 10 s, and the value was averaged by temperature history of each duration of 10 s following each time point.

### Cell culture

MCF-7 cells (RIKEN BRC, Saitama, Japan) and NHDFs (Cosmo Bio Co., Ltd., Tokyo, Japan) were cultured in Dulbecco’s Modified Eagle’s Medium (DMEM) (11965092; Thermo Fisher Scientific Inc.) supplemented with 10% fetal bovine serum (FBS) (Funakoshi Co., Ltd., Tokyo, Japan) and 1% penicillin (15140122; Thermo Fisher Scientific Inc.) at 37 °C under 5% CO_2_. Cell detachment was performed using 0.05% Trypsin–EDTA (25300; Life Technologies, Carlsbad, CA, USA).

### Live cell counting

To measure the live cell numbers, an automatic cell counter (TC20TM Automated Cell Counter; Bio-Rad, Hercules, CA, USA) was used, and trypan blue assay was employed to evaluate cell viability.

### RT-qPCR analysis

Relative mRNA expression was measured by reverse-transcription quantitative polymerase chain reaction (RT-qPCR) using total RNA extracted from the cells cultured for 24 h after stimulation. The mRNAs of the *BAX*, *BCL2*, *HSPA1A*, and *ACTB* genes were assessed, which encode the proteins BAX, BCL2, HSP70, and β-actin, respectively. Total RNA was extracted using a NucleoSpin RNA (740955.50; Takara Bio Inc., Tokyo, Japan) and quantified using a Thermal Cycler Dice Real Time System Lite (TP700; Takara Bio Inc.). RNA was reverse-transcribed into cDNA with PrimeScript Master Mix (Perfect Real Time) (RR036A; Takara Bio Inc.), an oligo(dT) primer, and random hexamer primers for 15 min at 37 °C and then 5 s at 85 °C. The cDNA concentration was quantified using a Biophotometer (6131; Eppendorf, Hamburg, Germany) and then decreased with RNase-free water (9012; Takara Bio Inc.) to 10 ng/µL cDNA. RT-qPCR was conducted in the Thermal Cycler Dice Real Time System Lite for 30 s at 95 °C and then 60 cycles of 5 s at 95 °C and 30 s at 60 °C. Each RT-qPCR contained 12.5 µl of TB Green Premix Ex Taq II (Tli RNaseH Plus) (RR820A; Takara Bio Inc.), 20 ng of cDNA, 0.4 µM of each of the forward and reverse primers, and 8.5 µL of RNase-free water. The primer sequences are shown in Table 1. RT-qPCR was performed in technical triplicates for each primer pair and cDNA sample. Furthermore, the reactions were conducted as biological triplicates under similar conditions. To confirm that the primer dimers were not responsible for the obtained fluorescence signals, melting curve analysis of amplicons was performed for each primer pair. Negative control reactions without the templates were also performed to ensure the data quality. Relative mRNA expression was normalized to β-actin mRNA and then calibrated using the quantity relative to the quantity obtained from cells detached by the conventional method. The fold change was calculated using the 2^−ΔΔCt^ method, in which C_t_ is the threshold cycle^14^.

**Table 1:** RT-qPCR primer sequences in this study.

Gene name	Gene bank number	Sequence(5′–3′)	Tm (℃)	Product size (bp)
*ACTB*	NM_001101.5	Forward TGGCACCCAGCACAATGAA	69.0	186
		Reverse CTAAGTCATAGTCCGCCTAGAAGCA	65.4	
*BAX*	NM_138761.4	Forward CTCAGGATGCGTCCACCAA	67.5	83
		Reverse CCTCTGCAGCTCCATGTTACTGTC	68.1	
*BCL2*	NM_000633.2	Forward AACATCGCCCTGTGGATGAC	67.3	146
		Reverse AGAGTCTTCAGAGACAGCCAGGAG	66.3	
*HSPA1A*	NM_005345.6	Forward CCTGGAGTCCTACGCTTCAAC	68.0	104
		Reverse CTTGACACTTGTCCAGCACCTTC	67.1	

### Immunofluorescent staining

Immunofluorescence was conducted for staining HSP, actin, and the nucleus. HSP was stained with Anti-HSP 70 (ADI-SPA-810-F; Enzo Life Sciences, Inc., Farmingdale, NY, USA) and Alexa Fluor 555-conjugated goat anti-rabbit IgG H&L (ab150078; Abcam, Cambridge, UK). Actin and the nucleus were stained with ActinGreen 488 Ready Probes reagent (R37110; Thermo Fisher Scientific Inc.) and Hoechst 33342 (H1399; Thermo Fisher Scientific Inc.). Staining was performed by the following procedure. First, 4% paraformaldehyde (09154-56; Nacalai Tesque, Inc., Kyoto, Japan) supplemented with Triton X-100 (C0875; Sigma-Aldrich) was applied to the cells, which were then incubated for 8 min at room temperature for fixation and permeabilization. Then, after blocking nonspecific binding sites with ImmunoBlock (CTKN001; KAC Co., Ltd., Kyoto, Japan) for 1 h at room temperature, ActinGreen 488 Ready Probes reagent and Hoechst 33342 were applied. Subsequently, the cells were incubated with 2 µL/1 mL Anti-HSP 70 for 1 h at room temperature. Then, they were incubated with Alexa Fluor 555-conjugated goat anti-rabbit IgG H&L for 30 min.

### Statistical analysis

The statistical significance of differences was evaluated by Welch’s t-test and ANOVA with multiple comparisons using Ryan’s method.* P* < 0.05 was accepted as statistically significant.

## Results

### Evaluation of cell culture system

The thermal stimulation system was composed of a metallic culture vessel and temperature regulation component, as shown in Fig. 1B, and therefore evaluations from the perspectives of mechanical engineering and biocompatibility were performed for each component.

To evaluate the effect of the culture vessel on cell culture, the morphology of the culture surface and the viability of cells cultured in the vessel were evaluated. To fabricate the metallic culture vessel, after the counterboring process, FPP was performed. As a result, spiral machining marks due to the counterboring process shown in Fig. 2A disappeared (Fig. 2C). Figure S2 shows the mean surface roughness (R_a_) and the arithmetic mean height of the surface profile (R_z_). Furthermore, 3.0 × 10^5^ cells were cultured for 24 h in the metallic dish and a 35-mm conventional culture dish to measure the biocompatibility of the vessel, resulting in our fabricated vessel showing good biocompatibility (Fig. 2D).

**Figure 3:**
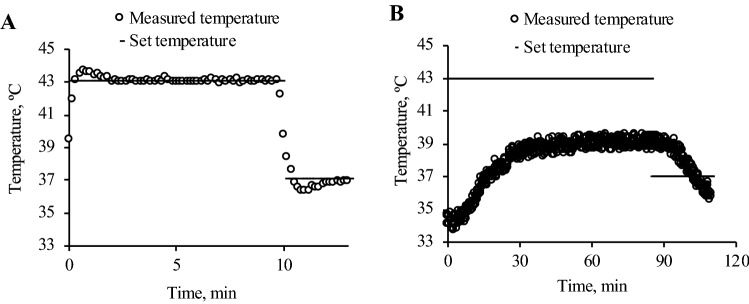
Our developed system immediately regulates temperature (**A**), while a conventional culture polymer dish shows moderate temperature change in its temperature history (**B**).

To evaluate the ability to control the temperature of the system, temperature histories of the culture surfaces were measured, as shown in Fig. 3. This indicated that the thermal stimulation caused by our system is more able to closely follow the target temperature than with a conventional culture vessel. This is because an incubator has its own temperature sensor. Due to the immediate temperature increment, the value indicated by the first plot shows the higher value than 37 °C. Note that the x-axes showing the time in each condition in Fig. 3 have different scales. We also determined whether there was a temperature distribution on the culture surface of the metallic culture vessel, as shown in Fig. S3. No uneven distribution of temperature was identified on the culture surface, resulting in the cells being exposed to homogeneous thermal stimulation. This demonstrated that the developed system can expose cells to thermal stimulation at a temperature that is immediately changed to the target value.

Convection in the medium due to the heating of the vessel was evaluated using fluorescent fine particles (FA-207; Sinloihi Co., Ltd., Kanagawa, Japan). Almost no convection was observed even with a set temperature of 47 °C, which was the maximum temperature employed in our study.

### Evaluation of thermal cytotoxicity in normal and cancer cells

To measure the thermal cytotoxicity in cells, thermal stimulations with several conditions were applied to the cells. The temperature and duration of the thermal stimulation were varied. To compare the tolerance of cancer and normal cells, MCF-7 cells and NHDFs were exposed to each thermal stimulus. The procedure of the experiment is shown in Fig. 1C. 3.0 × 10^5^ cells were cultured for 24 h in the metallic dish, and they were exposed to the thermal stimulation with the certain condition. Then, after following 30-min culture, culture medium was replaced and cells were culture for 24 h. Each assay was performed after 30-min culture or after finishing every procedure.

**Figure 4:**
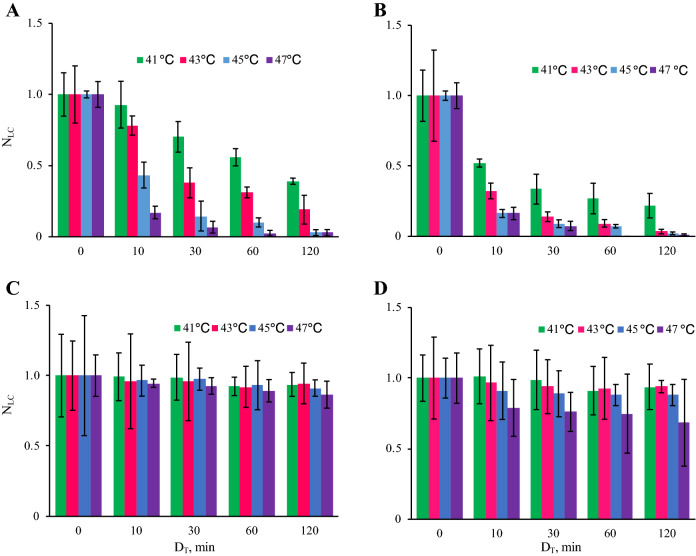
The number of live cells under each condition employed in our experiment. The viability of cancer cells decreased with increasing temperature and stimulus duration. The results of MCF-7 cells with culture for 30 min (**A**) and 24 h (**B**) after thermal stimulation and the results of NHDFs with culture for 30 min (**C**) and 24 h (**D**) after thermal stimulation (*n* = 4, mean ± SD). N_LC_: The live cell number normalized to the live cell number without thermal stimulation. D_T_: Duration of the thermal stimulation. The statistical analysis was performed with every temperature condition and the results are shown in Fig. S4. Note that no statistically significant difference was found in the results of NHDFs.

Figure 4 shows the thermal cytotoxicity in MCF-7 cells and NHDFs. This figure shows that cancer cells get stronger thermal cytotoxicity than normal cells, which explains the beneficial therapeutic effects of hyperthermia. Higher temperature and longer duration of thermal stimulus decreased the viability of cells, and this trend on the cancer cell viability was similar between 30-min and 24-h cultures. The results of statistical analysis of MCF-7 data between each duration of thermal stimulus are shown in Fig. S4. For NHDFs, after 30-min culture, the viabilities of cells did not show a clear trend for each condition. However, although there was no statistically significant difference between each condition, the viability of the cells after 24-h culture showed a certain trend where the live cell number decreased with increasing temperature.

Moreover, other than MCF-7 cells and NHDFs, two more cell models were employed and exposed to the thermal stimulation of 43 °C for 30 min (see Supplementary Note 1 and Fig. S5). One is cancer cells (A549), and the other is normal cells (HUVECs). As results, cancer cell models have shown the decrement in cell numbers, which indicates that cancer cells get stronger thermal cytotoxicity to the same thermal stimulation than normal cells.

### Glucose consumption and lactate production

**Figure 5:**
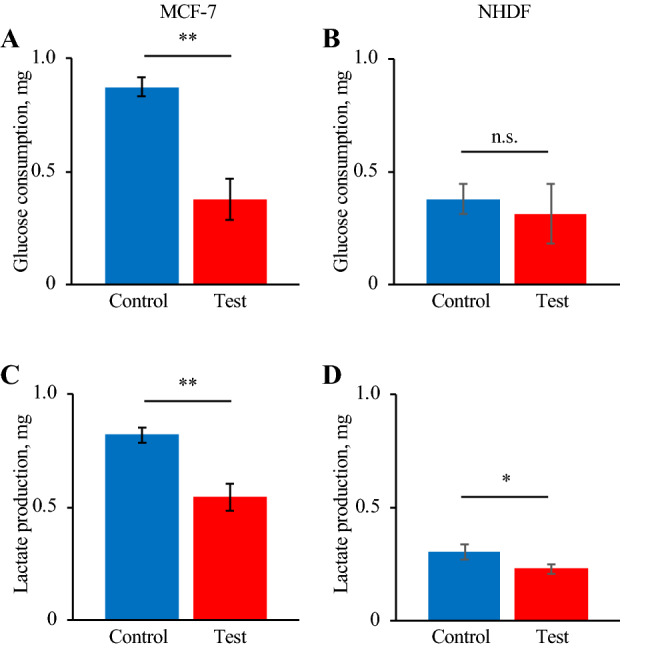
The indexes of glucose metabolism for 24 h of culture after thermal stimulus at 43 °C for 30 min. Glucose consumption of MCF-7 cells (**A**) and NHDFs (**B**) and lactate production of MCF-7 cells (**C**) and NHDFs (**D**) (n = 4, mean ± SD).

Glucose consumption and lactate production during 24-h culture after thermal stimulation were measured, as shown in Fig. 5. This figure shows that cancer cells dramatically decreased their glucose consumption, while there was little decrease in normal cells. This result is consistent with the live cell numbers.

### Apoptosis-related genes

**Figure 6:**
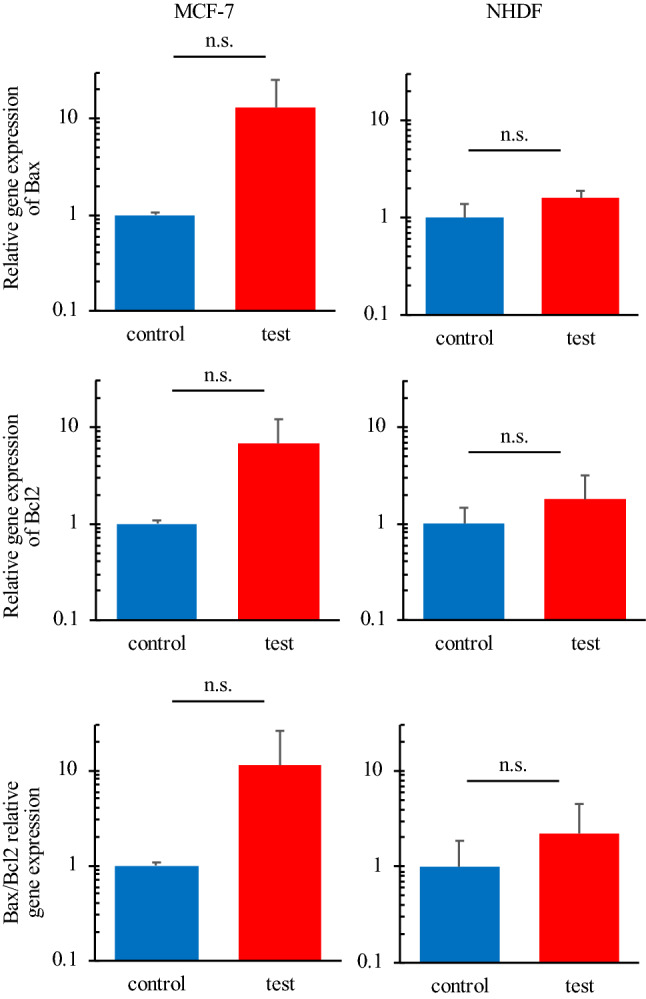
Relative mRNA expression levels of apoptosis-related genes. Cancer cells showed more prominent apoptosis than normal cells (*n* = 3, mean ± SD).

Figure 6 shows the RT-qPCR results on the relative mRNA expression levels of apoptosis-related genes. The RT-qPCR assay was performed with the cells cultured for 24-h after thermal stimulation at 43 °C for 30 min to quantify the relative expression of *BAX* and *BCL2*. As shown in Fig. 6, both *BAX* and *BCL2* were strongly activated in cancer cells due to the thermal stimulus, while they were not in normal cells. Furthermore, the ratio of *BAX*/*BCL2*, which is an index of apoptosis promotion, also differed dramatically between cancer and normal cells after thermal stimulation^15^.

### Expression of HSP

**Figure 7:**
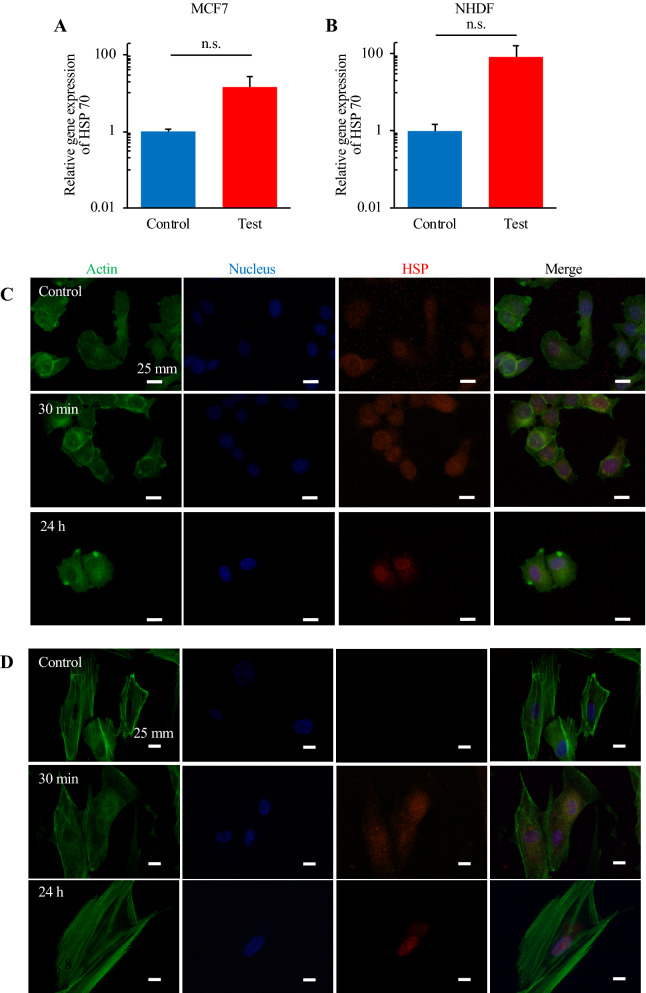
Comparison of the expression of HSP70 in MCF-7 cells (**A** and **C**) and NHDFs (**B** and **D**) after thermal stimulation. RT-qPCR assay (**A** and **B**) (n = 3, mean ± SD) and immunofluorescent staining (**C** and **D**) were performed. Actin, the nucleus, and HSP were stained green, blue, and red, respectively.

Figure 7 shows the different trends of HSP expression at mRNA and protein levels between cancer and normal cells. Both cancer and normal cells showed the increased mRNA expression of *hspa1a*, with this increase being greater in normal cells. In contrast, at the protein level, the cells showed completely different trends. Cancer cells expressed HSP even without thermal stimulus, and the expression of HSP did not dramatically change even after thermal stimulus. However, while normal cells did not initially show HSP expression, 30 min after stimulus they started to express it. Furthermore, it was demonstrated that HSP localized to the nucleus of normal cells upon 24-h culture after thermal stimulus.

## Discussion

In this study, we designed a novel culture system capable of regulating the culture temperature immediately. This invention is largely based on the metallic culture vessel because metallic material has higher thermal conductivity than the materials conventionally used for cell culture scaffolds, such as polystyrene^16–19^. Stainless steel (316L) was used for this metallic culture vessel, given its high biocompatibility and processability^20^. Culture-dish-like vessels were fabricated via counterboring shown in Fig. 2A and FPP. Figure 2B shows the metallic culture vessel fabricated. As presented in previous studies, when there is a structural pattern on the culture surface, cells can be oriented along with such patterns. Furthermore, the roughness of the surface without FPP is sufficient to confer a particular orientation to cells, as shown in Fig. S2^21^. Because the morphology of adhered cells affects cell activity, homogeneous culture surfaces should be obtained to perform accurate analyses of the thermal cytotoxicity or other effect from thermal stimulation on cells^9, 22, 23^. Owing to FPP, however, the processing marks of counterboring disappeared (Fig. 2C). Note that culture surfaces subjected to FPP are adequate for cell culture^12, 24^. Furthermore, from the perspective of biocompatibility, as shown in Fig. 2D, during the 24-h culture in a conventional culture dish and the metallic vessel, no differences in the viability and proliferation of cells were observed. Thus, we concluded that the stainless steel (316L) culture vessel developed in this study can be employed for the culture system to investigate cell function.

This paper reports development of a culture system makes it possible to systematically investigate the thermal cytotoxicity in cells. Our novel system has a cooling system that achieves a dramatic decrement of temperature and can purely provide thermal stimulus to cells without other mechanical stimulation. Note that the lack of convection flow in our system has already been confirmed. It has been reported that HSP, which confers thermotolerance to cells, can also be expressed by mechanical stimulation rather than heat shock^25^. Alternatively, it was recently reported that combinations of hyperthermia and other stimuli enhanced the cell killing effect^10, 15, 26, 27^. On the other hand, in previous studies, owing to the lack of a system that achieves immediate temperature regulation, there was a gap between the set temperature and the actual realized culture temperature to which cells were exposed. As reported in previous studies, a certain duration has been required to attain the set temperature, as also confirmed in Fig. 3B^28^. It has been asserted that, during the mild temperature increase that occurs in such systems, cells attain thermotolerance^29^. Thus, immediate temperature regulation including temperature increasing and decreasing is very important for investigating the actual thermal cytotoxicity to an actual condition of thermal stimulation in original cells. To date, technologies employing the photo-thermal effect, ultrasound, radio frequency, and magnetic nanoparticles, for example, for an immediate temperature increment have been reported, although these methods offer no way of decreasing the temperature^30–33^. Other problems also emerged with these approaches. For example, a homogeneous temperature distribution on the cells would be difficult to achieve by methods using the photo-thermal effect and ultrasound^33^. Additionally, in the methods using ultrasound and magnetic particles, cells are potentially affected by mechanical stimuli other than thermal stimulation, resulting in altered cell functions^30, 34–38^. Note that mechanical stimulation induces the expression of HSP^25^. Moreover, radio frequency can affect cell conditions and functions due to the altered electrical field^39^. Thus, we concluded that our system provides the only solution for investigating the thermal cytotoxicity in original cells.

For the above reasons, for comparison with our study, we excluded studies using an immediate temperature increment with other forms of stimulations. Thus, we compared the results in our study to the data obtained in studies using conventional and simple temperature regulation methods with an incubator or a water bath^8, 27, 40, 41^. Figure 4 shows the thermal cytotoxicity, which is qualitatively similar but quantitatively different to that in previous reports. Although cell reactions to certain stimulations including thermal cytotoxicity depend on the culture environment and the technician who handled the cells, the developed system for applying a thermal stimulus is also one of the most influential factors^42, 43^. The different results reported in previous studies can be explained from two perspectives. One is simply that there are differences in the temperature and duration of the thermal stimulus that cells were exposed to among previous studies and our study, which was indicated in Fig. 3. The other reason is more complex. As already mentioned, in previous studies, the temperature of the thermal stimulus was gradually increased, which confers cells with thermotolerance. We thus argue that the thermal cytotoxicity in cells shown in our study is very accurate, and our study is the first to investigate the *original* thermal cytotoxicity in cells.

There is a reported algorithm called cumulative equivalent minutes algorithm, which is capable of predicting the effect of thermal dose from temperature history^44^. This might be helpful to investigate the simple thermotolerances of cancer cells. However, especially for normal cells, which actively expressed HSP with thermal stimulation, there may be a gap between the prediction and the actual experimental results, since HSP confer the thermotolerance to cells. Further, in future works, our system can be combined with other stimulation for investigation of effective therapies or other fundamental studies. Thus, actual experimental setup to regulate thermal stimulation on cells has been required other than simple prediction.

We focused on one set of conditions of thermal stimulus (43 °C/30 min) as a good arrangement for applying hyperthermia, and performed several assays for both MCF-7 cells and NHDFs. Cell metabolisms were evaluated by glucose consumptions and lactate productions to confirm if there was no abnormal effect of thermal stimulation especially on normal cells’ metabolism. If a huge variation was reported especially on normal cells, thermal stimulation should have some effect on healthy cells, which indicates the risk of hyperthermia. There was, actually, no abnormal increase in glucose consumption or lactate production in normal cells (Fig. 5), which indicates that these cells were not transformed into another state, such as a cancerous one^45^. Actually, although there was a certain decrement, it is natural since there was also a small decrement in the number of live cells (Figs. 4 and S5). Further the trend of the results of glucose consumption and lactate production assays (Fig. 5) corresponded to the findings on the number of live cells (Figs. 4 and S5). This supported the finding that cancer cells dramatically decreased in number while normal cells showed only a small decrease. The above discussion supports the assertion that the hyperthermia with the certain condition of thermal stimulation had very few side effects. The findings on the expression of genes involved in apoptosis also corresponded to the results of live cell numbers. Figure 6 indicates that the cancer cells were in the state of apoptosis, in contrast to the normal cells, because the ratio of *BAX*/*BCL2* used as an index of apoptosis was dramatically changed in cancer cells due to the thermal stimulus^15^. As shown in Fig. 4, the decrease in the number of live cancer cells was clearer after 24-h culture than immediately after thermal stimulus. Apoptosis has been reported to require a few hours to occur, while necrosis occurs within a short time^46^. In contrast, normal cells strongly expressed HSP upon the thermal stimulus, which confers thermotolerance, as shown by the quantification of mRNA (Fig. 7A and B). Furthermore, Fig. 7C and D show that the different trend of HSP expression and localization between cancer cells and normal cells. In cancer cells, HSP70 were shown without thermal stimulation and were not localized at any specific position. On the other hand, while normal cells did not show any HSP70 expression without thermal stimulation, they started expressed HSP70 30 min later after the thermal stimulation and showed the localization of the protein at a nucleus 24 h later after thermal stimulation. The localization of HSP and its import into the nucleus are key phenomena in obtaining thermotolerance, but it has been reported that, in both cancer and normal cells, HSP localization at the nucleus occurred after thermal stimulus^47^. The present work is the first to clearly show the difference of HSP localization between cancer and normal cells (see Fig. 1A). Although the key factor behind this remains unclear, we predict that the system achieving immediate temperature regulation may be a key.

In conclusion, in the present work, we developed a cell culture system enabling systematic investigation of the thermal cytotoxicity in cells. This is due to the immediate temperature regulation achieved in our system. We believe that the developed system will become fundamental equipment for in vitro studies of hyperthermia, which is one of the promising candidates to physically kill cancerous cells without any side effect. Our developed system, actually, shows a different and novel result with similar experiments in previous researches. Further, since the trend that the thermal cytotoxicity in cancer cells are more outstanding than normal cells has been confirmed with plural cell types as shown in Fig. S5, it has been demonstrated that our method can have a versatility. From the data shown in this paper, many works can be suggested; we can use other thermal conditions, other cell species, or even tumor models for instance. Other than the difference of cell species, it has been known that cancer cells composing a tumor model has a different function, which should be a very interesting research topic^48^. We can utilize reported three-dimensional culture methods for cancer cells or normal cells to mimic tissue models, and the present culture system can be combined with such tissue culture methods in our future works^49, 50^. Even at 24 h after thermal stimulation, the cancer cells still showed a tendency for undergoing apoptosis (Fig. 6), which indicates that further culture may cause more apoptosis. Moreover, HSP was localized at the nucleus in normal cells, while the mRNA expression of *hspa1a* was upregulated even at 24 h after thermal stimulation. This indicates that normal cells had thermotolerance at this point in time. Thus, it could be predicted that we could apply more intense thermal stimulation for hyperthermia treatment after the first thermal stimulation. Because such localization disappears after a certain amount of time, the duration for which the HSP localization is maintained and thermotolerance is shown should be measured^9^. Finally, the scope of the application of our system should not be limited to fundamental studies of hyperthermia, but expanded to other bioengineering studies. The effect of culture temperature was investigated to achieve a better culture protocol of mass culture or for tissue engineering and regenerative medicine, for example^34, 51^. Thus, this study could promote research across the whole field of bioengineering.

## Supplementary Information

Supplementary Information.

## Data Availability

Data supporting the findings of this study are available in the article and Supplementary information files, or from the corresponding author upon request.
