# Supplementary material for: Development of accurate temperature regulation culture system with metallic culture vessel demonstrates different thermal cytotoxicity in cancer and normal cells
Source: Sci Rep. 2021 Nov 2;11:21466. doi: 10.1038/s41598-021-00908-0 (PMC8563756; doi:10.1038/s41598-021-00908-0)
Supplement: Supplementary file 1 — Supplementary Information. [file 41598_2021_908_MOESM1_ESM.pdf]

Development of accurate temperature regulation culture system with metallic culture vessel demonstrates different thermal cytotoxicity in cancer and normal cells

Chikahiro Imashiro[1,2*], Haruka Takeshita[2], Takashi Morikura[2], Shogo Miyata[2], Kenjiro Takemura[2], & Jun Komotori[2*]

[1] Institute of Advanced Biomedical Engineering and Science, Tokyo Women's Medical University, TWIns, Tokyo 162-8666, Japan [2]Department of Mechanical Engineering, Keio University, Yokohama 223-8522, Japan. Correspondence and requests for materials should be addressed to C.I. and J.K. (email: imashiro.chikahiro@twmu.ac.jp, komotori@mech.keio.ac.jp).

Supplementary Note 1

To investigate the difference of thermal cytotoxicity on normal and cancer cells with the same thermal stimulation, human cell line derived from lung cancer (A549) and human umbilical vein endothelial cells (HUVECs) were utilized as model cells other than MCF-7 and NHDF. A549 (RIKEN BRC, Saitama, Japan) and HUVEC (C2519A; Lonza, Basel, Switzerland) were cultured in DMEM supplemented with 10% FBS and 1% penicillin and endothelial growth medium and supplements (CC－3162; Lonza) with respectively at 37°C under 5% CO_2. Cell detachment was performed using 0.05% Trypsin-EDTA. For the experiment, they were cultured in the same procedure with MCF-7 and NHDF and exposed to the thermal stimulation with the certain condition (43°C/30 min). The viability of cells was evaluated with 30 min culture after the thermal stimulation. The comparison of live cell ratio of each cell type was shown in Fig. S5. As shown in the figure, thermal cytotoxicity on cancer cells is stronger than normal cells with the same thermal stimulation.

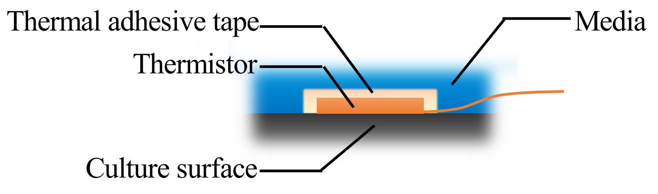

Fig. S1 Schematic image of the way to adhere the thermistor on culture surface with thermal adhesive tape.

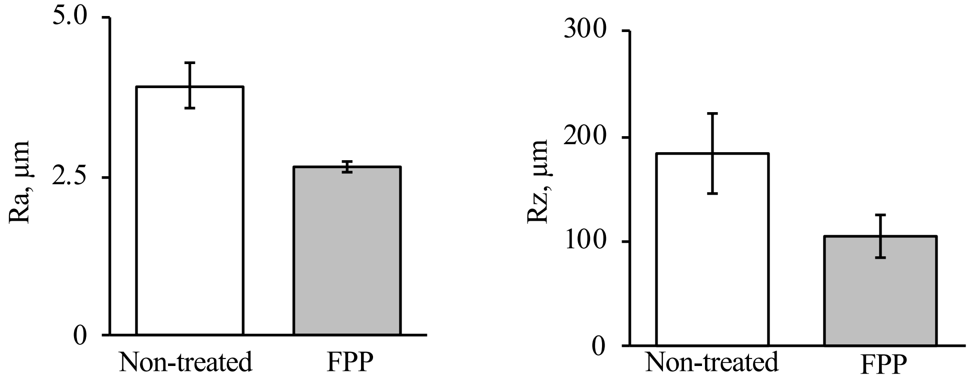

Fig. S2 Measured Ra and Rz of untreated and FPP samples of the surfaces of fabricated metallic vessels (n = 10 and 6 for untreated and FPP samples, respectively).

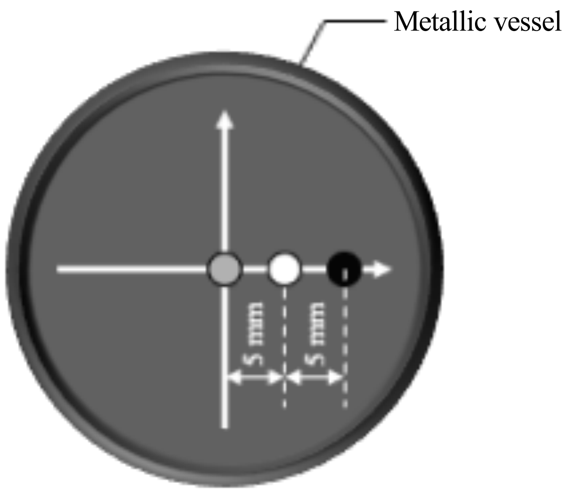

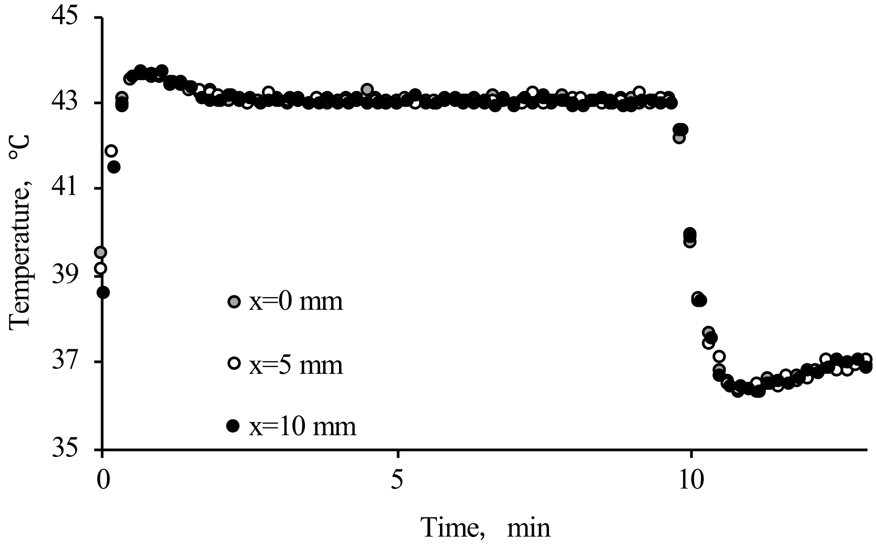

Fig. S3 Temperature history of each location on the culture surface of the fabricated vessel. The employed temperature regulation conditions are the same as in Fig. 3. For the measurement, we used four sensors for this evaluation at once. One of them was connected to temperature regulation system, while the others were used to evaluate the temperature distribution.

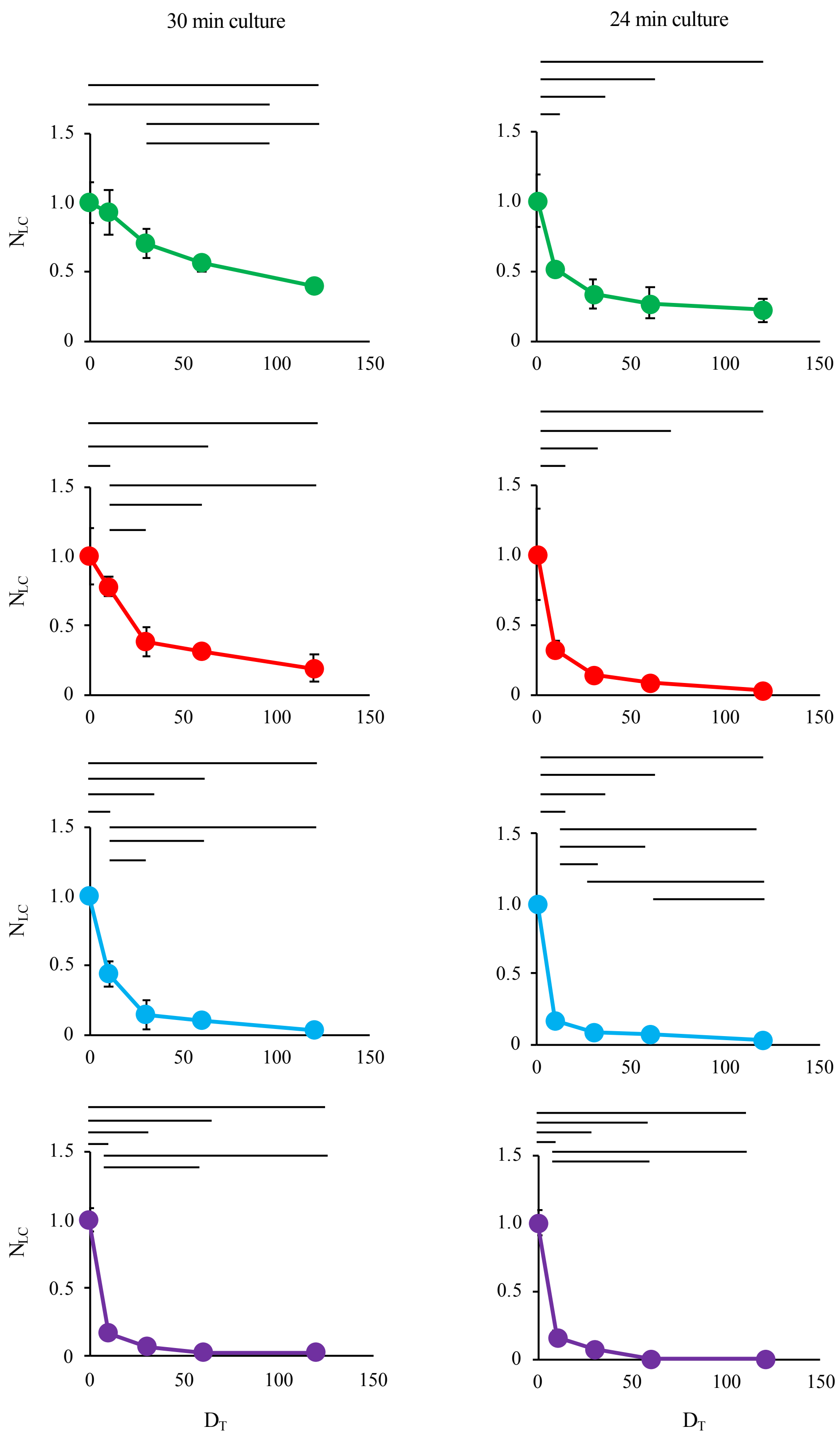

Fig. S4 Results of statistical analysis on the number of live MCF-7 cells (n = 4, mean ± SD, P<0.05). We employed a scatterplot graph to show the relationship between DT and NLC. NLC: The live cell number normalized to the live cell number without thermal stimulation. DT: Duration of the thermal stimulation.

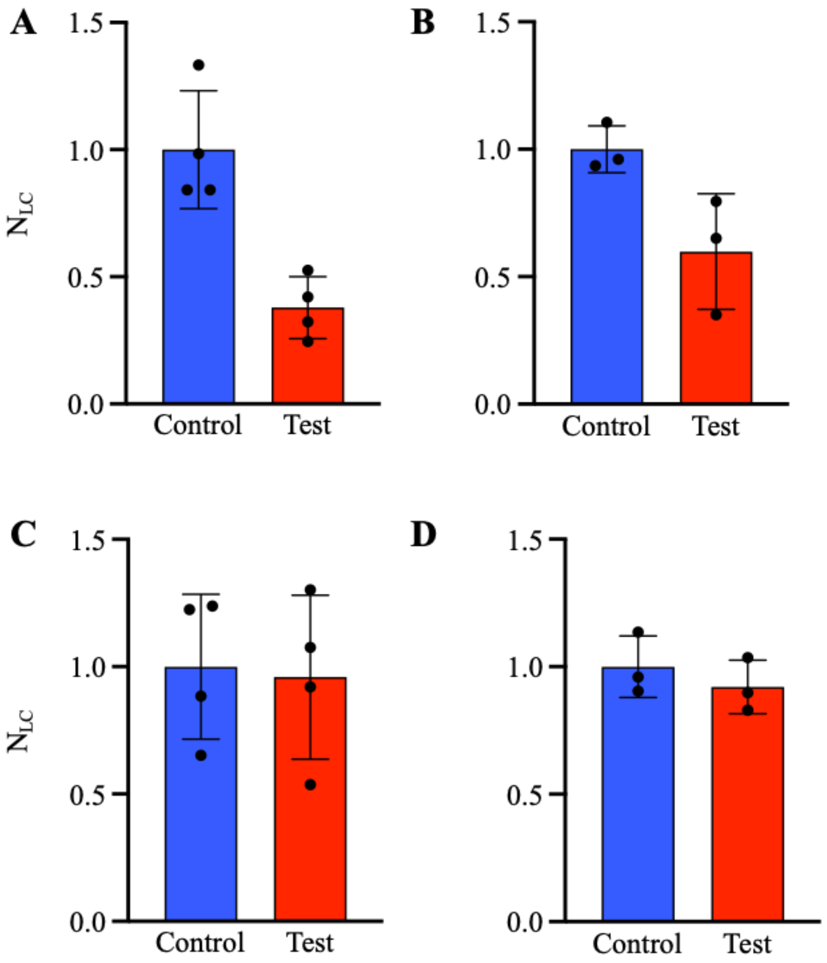

Fig. S5 Comparison of thermal cytotoxicity in each cell type in response to the thermal stimulation of 43°C for 30 min. As cancer cell models, MCF7 (A) and A549 (B) were utilized, while as normal cell model, NHDF (C) and HUVECs (D) were utilized (mean ± SD). N_{LC}: The live cell number normalized to the live cell number without thermal stimulation. Cancer cell models show the decreased N_{LC} due to the thermal stimulation. On the other hand, there is not such a clear trend in normal cell models.

Table S1. Conditions for fine particle peening.

Particle	Al_2O_3 #1500
Pressure	0.6 MPa
Distance between the nozzle and material	150 mm
Peening time	60 s
